# Angiogenic T cell expansion correlates with severity of peripheral vascular damage in systemic sclerosis

**DOI:** 10.1371/journal.pone.0183102

**Published:** 2017-08-10

**Authors:** Mirko Manetti, Sara Pratesi, Eloisa Romano, Silvia Bellando-Randone, Irene Rosa, Serena Guiducci, Bianca Saveria Fioretto, Lidia Ibba-Manneschi, Enrico Maggi, Marco Matucci-Cerinic

**Affiliations:** 1 Department of Experimental and Clinical Medicine, Section of Anatomy and Histology, University of Florence, Florence, Italy; 2 Department of Experimental and Clinical Medicine, Section of Internal Medicine, Azienda Ospedaliero-Universitaria Careggi (AOUC), University of Florence, Florence, Italy; Keio University, JAPAN

## Abstract

The mechanisms underlying endothelial cell injury and defective vascular repair in systemic sclerosis (SSc) remain unclear. Since the recently discovered angiogenic T cells (Tang) may have an important role in the repair of damaged endothelium, this study aimed to analyze the Tang population in relation to disease-related peripheral vascular features in SSc patients. Tang (CD3^+^CD31^+^CXCR4^+^) were quantified by flow cytometry in peripheral blood samples from 39 SSc patients and 18 healthy controls (HC). Circulating levels of the CXCR4 ligand stromal cell-derived factor (SDF)-1α and proangiogenic factors were assessed in paired serum samples by immunoassay. Serial skin sections from SSc patients and HC were subjected to CD3/CD31 and CD3/CXCR4 double immunofluorescence. Circulating Tang were significantly increased in SSc patients with digital ulcers (DU) compared either with SSc patients without DU or with HC. Tang levels were significantly higher in SSc patients with late nailfold videocapillaroscopy (NVC) pattern than in those with early/active NVC patterns and in HC. No difference in circulating Tang was found when comparing either SSc patients without DU or patients with early/active NVC patterns and HC. In SSc peripheral blood, Tang percentage was inversely correlated to levels of SDF-1α and CD34^+^CD133^+^VEGFR-2^+^ endothelial progenitor cells (EPC), and positively correlated to levels of vascular endothelial growth factor and matrix metalloproteinase-9. Tang were frequently detected in SSc dermal perivascular inflammatory infiltrates. In summary, our findings demonstrate for the first time that Tang cells are selectively expanded in the circulation of SSc patients displaying severe peripheral vascular complications like DU. In SSc, Tang may represent a potentially useful biomarker reflecting peripheral vascular damage severity. Tang expansion may be an ineffective attempt to compensate the need for increased angiogenesis and EPC function. Further studies are required to clarify the function of Tang cells and investigate the mechanisms responsible for their change in SSc.

## Introduction

Systemic sclerosis (SSc) is an orphan connective tissue disease characterized by early generalized microvascular damage and specific immunologic abnormalities evolving into fibrosis of the skin and internal organs [1–3]. In SSc, detailed assessment of skin microcirculation by nailfold videocapillaroscopy (NVC) allows the detection of a variety of morphological changes reflecting the severity and progression of peripheral microvascular injury, including giant capillaries, microhemorrhages, loss of capillaries with the appearance of avascular areas, and abnormal capillary shapes evocative of disturbances in post-ischemic vascular repair and growth [2, 4, 5].

Although the mechanisms underlying endothelial cell damage and defective repair remain incompletely understood, a large body of evidence suggests that a profound impairment of either neoangiogenesis or endothelial progenitor cell (EPC)-driven vasculogenesis is a key feature in SSc [6–10]. In fact, the dysregulation of multiple cellular and molecular pathways, that are required for post-ischemic endothelial cell and EPC compensatory responses, prevents peripheral vascular recovery [2, 11–16]. This often results into severe peripheral vascular manifestations such as digital ulcers (DU) and gangrene [2].

Recent studies suggest that a specific T cell population, termed angiogenic T cells (Tang), may promote the formation of new blood vessels and enhance the repair of damaged endothelium [17]. Tang cells are characterized by the co-expression of CD3, platelet-endothelial cell adhesion molecule-1 (CD31), and the receptor for the CXC chemokine stromal cell-derived factor-1 (SDF-1)/CXCL12 (CXCR4 or CD184) [17, 18]. *In vitro* experiments showed that Tang cells may foster postnatal vasculogenesis and endothelial repair by stimulating early EPC differentiation and endothelial cell proliferation and functions possibly through the secretion of high levels of proangiogenic factors, such as vascular endothelial growth factor (VEGF), interleukin (IL)-8, IL-17 and matrix metalloproteinase (MMP)-9 [17]. Indeed, it has been demonstrated that CD3^+^CD31^+^CXCR4^+^ Tang cells constitute the central cluster of EPC colonies during cultures of human peripheral blood mononuclear cells, and that Tang depletion could abrogate EPC differentiation and functionality [17]. Moreover, *in vivo* studies also highlighted the relevance of the Tang cell subset in the process of new capillary formation in a mouse model of hind limb ischemia [17]. Of note, it has been recently reported that altered circulating Tang cell frequencies may be associated with cardiovascular disease in rheumatoid arthritis (RA), systemic lupus erythematosus (SLE) and antineutrophil cytoplasmic antibody (ANCA)-associated vasculitis [18–20].

On these premises, the aim of the present study was to investigate the Tang cell population and to verify its possible correlation with the peripheral vascular features of SSc.

## Materials and methods

### Patients and controls

Peripheral blood samples were obtained from 39 consecutive patients fulfilling the 2013 ACR/EULAR classification criteria for SSc [21] and 18 age-matched and sex-matched healthy controls (HC). Patients with SSc were further classified as limited cutaneous SSc (lcSSc; n = 24) or diffuse cutaneous SSc (dcSSc; n = 15) according to LeRoy *et al*. [22]. At the time peripheral blood was drawn, the presence of DU was recorded. NVC was performed on all 10 fingers by a single rheumatologist, and images were scored blindly by two experienced examiners who divided patients into three NVC patterns (*i*.*e*., early, active and late) [23]. At the time of sampling, patients were not on immunosuppressive medications, corticosteroids or other potentially disease-modifying drugs. Before blood sampling, they were washed out for 10 days from oral vasodilating drugs and for 2 months from intravenous prostanoids. Fresh venous blood samples were drawn and either immediately processed for flow cytometric analysis or left to clot for 30 minutes before centrifugation at 1,500 g for 15 minutes for serum separation. Serum samples were stored in aliquots at −80°C until used. Paraffin-embedded sections of lesional forearm skin biopsies were obtained from 7 patients with early dcSSc (disease duration <2 years from first non-Raynaud symptom) and 6 age-matched and sex-matched HC, as described elsewhere [24]. The study was conducted in compliance with the Declaration of Helsinki and was approved by the local institutional review board at the Azienda Ospedaliero-Universitaria Careggi (AOUC), Florence, Italy (AOUC 69/13). All subjects provided written informed consent.

### Flow cytometric analysis of Tang cells and EPC

Circulating Tang cells were characterized by flow cytometry. Briefly, 300 μl of fresh whole peripheral blood was stained with anti-CD3 allophycocyanin-H7 (APC-H7), anti-CD31 fluorescein isothiocyanate (FITC) and anti-CXCR4 phycoerythrin (PE) antibodies or isotype-matched control IgG antibodies (all from BD Biosciences, San Diego, CA, USA) according to the manufacturer's instructions. Subsequently, red blood cell lysis was performed and cells were analyzed. At least 30,000 CD3^+^ T lymphocytes events were acquired using a FACSCanto II flow cytometer (BD Biosciences) and analyzed using FACSDiva software (BD Biosciences). CD3^+^ T lymphocytes double-positive for CD31 and CXCR4 were considered Tang cells. For the evaluation of circulating EPC, CD34^+^ cells were enriched from peripheral blood mononuclear cells from SSc patients and HC using the CD34 MicroBead Kit (Miltenyi Biotec, Bergisch Gladbach, Germany) and stained with anti-CD34 FITC, anti-VEGF receptor-2 (VEGFR-2) Peridinin Chlorophyll Protein-Cy5.5 (PerCP-Cy5.5) (both from Miltenyi Biotec) and anti-CD133 PE (BD Biosciences) antibodies. At least 5,000 events in the CD34^+^ enriched population gate were acquired at low rate and EPC were identified as CD34^+^CD133^+^VEGFR-2^+^ cells.

### Absolute blood cell counts and evaluation of Tang cell phenotype

Trucount Tubes (BD Biosciences) were used for determining the absolute numbers of Tang cells in SSc patients and HC. Fresh whole peripheral blood (100 μl) was added into the Trucount Tube and then stained with anti-CD3 Pacific Blue, anti-CD8 PerCP-Cy5.5 (Miltenyi Biotec), anti-CD4 PE-Cy7, anti-CD31 FITC, anti-CXCR4 PE and anti-CD28 allophycocyanin (APC) antibodies (all from BD Biosciences). After 15 minutes, red blood cells were lysed with NH_4_Cl and then cells were analyzed using a FACSCanto II flow cytometer (BD Biosciences) equipped with FACSDiva software (BD Biosciences). The absolute cell count was obtained multiplying the number of positive cell events by the number of Trucount Tube beads and subsequently dividing by the number of Trucount bead events.

### Immunofluorescence staining of skin sections

For antigen retrieval, paraffin-embedded serial skin sections (5 μm thick) were deparaffinized and boiled for 10 minutes in 10 mM sodium citrate buffer (pH 6.0). After washing in phosphate buffered saline (PBS), the sections were incubated in 2 mg/ml glycine for 10 minutes to quench autofluorescence and then blocked for 1 hour at room temperature with 1% bovine serum albumin in PBS. The slides were subsequently incubated overnight at 4°C with a mixture of prediluted mouse monoclonal anti-CD3 (catalog number ab7507, Abcam, Cambridge, UK) and rabbit polyclonal anti-CD31 (1:50 dilution; catalog number ab28364, Abcam) or rabbit monoclonal anti-CXCR4 (1:100 dilution; catalog number ab124824, Abcam) antibodies. The day after, skin sections were extensively washed in PBS and incubated with a mixture of Alexa Fluor-488-conjugated goat anti-mouse IgG and Rhodamine Red-X-conjugated goat anti-rabbit IgG (both 1:200 dilution; Molecular Probes, Eugene, OR, USA) for 45 minutes at room temperature in the dark. Irrelevant isotype-matched and concentration-matched mouse and rabbit IgG (Sigma-Aldrich, St. Louis, MO, USA) were used as negative controls. Nuclei were counterstained with 4′,6-diamidino-2-phenylindole (DAPI) (Chemicon International, Temecula, CA, USA). The immunostained sections were then observed under a Leica DM4000 B microscope (Leica Microsystems, Mannheim, Germany). Fluorescence images were captured using a Leica DFC310 FX 1.4-megapixel digital color camera equipped with the Leica software application suite LAS V3.8 (Leica Microsystems).

### Determination of SDF-1α, VEGF, MMP-9, IL-8 and IL-17 serum levels

Levels of SDF-1α, VEGF, MMP-9, IL-8 and IL-17 in serum samples were measured by commercial quantitative colorimetric sandwich enzyme-linked immunosorbent assay (Human CXCL12/SDF-1α Quantikine ELISA Kit, Human VEGF Quantikine ELISA Kit and Human IL-17 DuoSet ELISA Kit, all from R&D Systems, Minneapolis, MN, USA; Human IL-8/NAP-1 INSTANT ELISA Kit and Human MMP-9 Platinum ELISA Kit, both from eBioscience, San Diego, CA, USA) according to the manufacturer’s instructions. The detection range was 0.156–10.0 ng/ml for SDF-1α, 31.2–2,000 pg/ml for VEGF, 0.23–15.0 ng/ml for MMP-9, 15.6–1,000 pg/ml for IL-8 and 15.6–1,000 pg/ml for IL-17. Serum samples were diluted 1:250 for the MMP-9 assay. Concentrations were calculated using a standard curve generated with specific standards provided by the manufacturer. Each sample was measured in duplicate.

### Statistical analysis

Data are expressed as the median and interquartile range (IQR). Differences between two independent groups were determined using the nonparametric Mann–Whitney U test. Correlations were evaluated by nonparametric Spearman’s rank correlation analysis. For all tests, a two-sided p-value less than 0.05 was considered significant. Data analyses were performed using SPSS 24.0 software (SPSS, Chicago, IL, USA).

## Results

**Fig 1:**
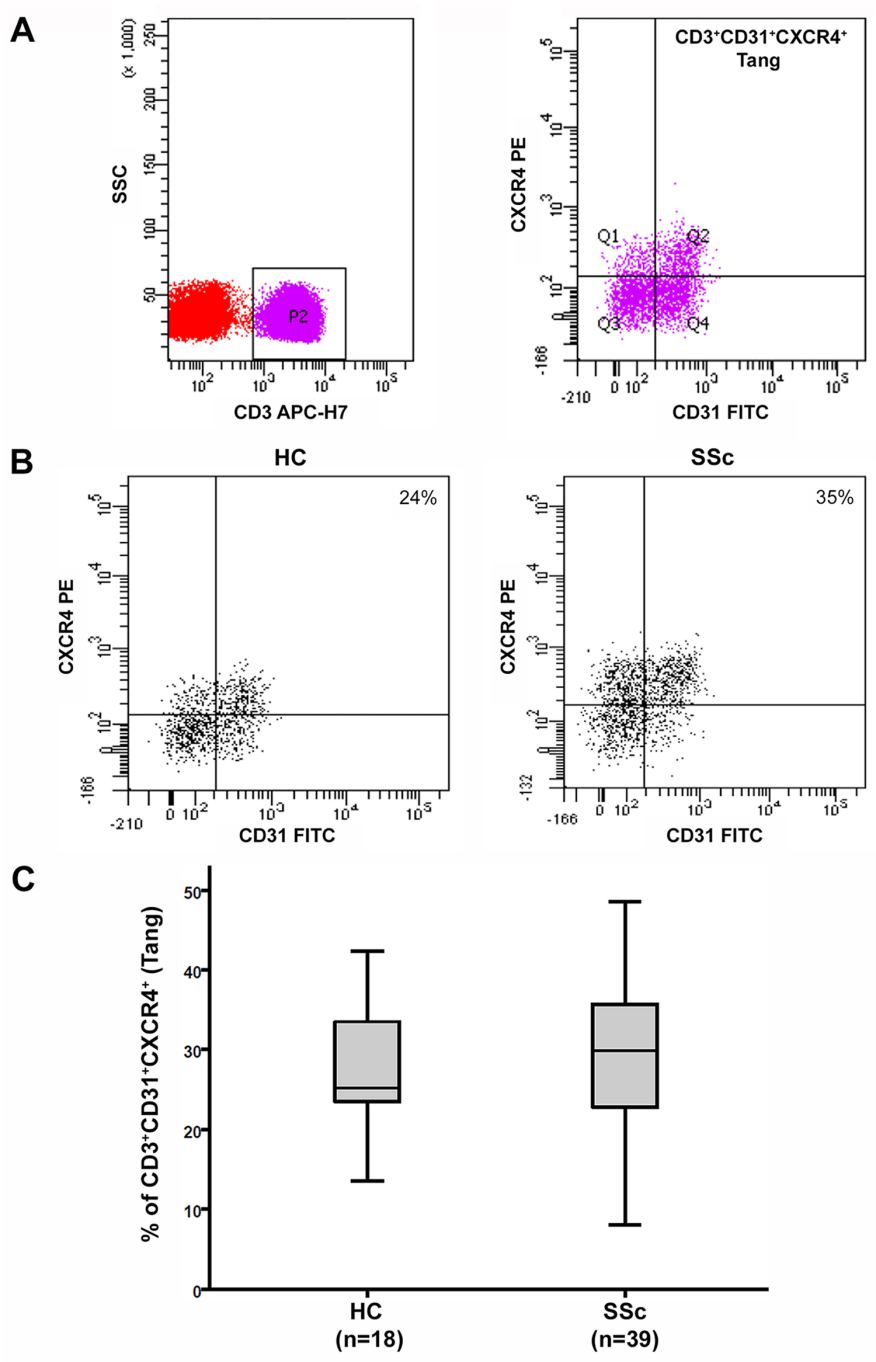
Angiogenic T cells (Tang) in peripheral blood of healthy controls (HC) and systemic sclerosis (SSc) patients. **(A)** Gating strategy used for the flow cytometric enumeration of Tang. Gated CD3^+^ T lymphocytes were analyzed for CD31 and CXCR4 expression by flow cytometry. Tang population was identified as CD3^+^CD31^+^CXCR4^+^ cells in the lymphocyte gate. Quadrants were set according to the fluorescence signal provided by the isotype controls. **(B)** Representative CD31 versus CXCR4 dot plots of a HC and a SSc patient. **(C)** Percentages of circulating CD3^+^CD31^+^CXCR4^+^ Tang cells in total CD3^+^ T cells from HC (n = 18) and SSc patients (n = 39). Data are shown as box plots. Each box represents the 25th to 75th percentiles. Lines inside the boxes represent the median. Lines outside the boxes represent the 10th and the 90th percentiles. Differences were evaluated by Mann–Whitney U test.

Demographic and clinical characteristics of SSc patients are summarized in Table 1. Peripheral blood samples from 39 SSc patients and 18 HC were analyzed by flow cytometry, quantifying Tang cell population by means of their CD3, CD31 and CXCR4 expression (Fig 1A and 1B).

**Table 1:** Demographic and clinical characteristics of patients with systemic sclerosis (SSc).

Age, mean ± SD (years)	51.8 ± 13.6
Male	5 (12.8%)
Female	34 (87.2%)
lcSSc subset	24 (61.5%)
dcSSc subset	15 (38.5%)
Disease duration, mean ± SD (years)*	6.8 ± 4.7
ANA	39 (100%)
ACA	20 (51.3%)
Anti-topo I	12 (30.8%)
Digital ulcers	18 (46.1%)
Early NVC pattern	6 (15.4%)
Active NVC pattern	14 (35.9%)
Late NVC pattern	19 (48.7%)
mRSS, mean ± SD	11.5 ± 5.7
ILD^§^	16 (41.0%)

ACA, anticentromere antibodies; ANA, antinuclear antibodies; Anti-topo I, anti-topoisomerase I antibodies; dcSSc, diffuse cutaneous systemic sclerosis; ILD, interstitial lung disease; lcSSc, limited cutaneous systemic sclerosis; mRSS, modified Rodnan skin score; NVC, nailfold videocapillaroscopy.

Except where indicated otherwise, values are the absolute number and percentage of patients.

*Disease duration was calculated since the first non-Raynaud’s symptom of SSc.

^§^Determined by high-resolution computed tomography scan.

The percentage of circulating CD3^+^CD31^+^CXCR4^+^ Tang cells in total CD3^+^ T cells was not different between the whole SSc patient cohort (median 29.9, IQR 22.3−36.2) and HC (median 25.2, IQR 23.3−33.5) (Fig 1C). No difference in circulating Tang cells was detected between patients with lcSSc (median 30.4, IQR 23.4−36.4) and those with dcSSc (median 28.3, IQR 21.0−36.2).

**Fig 2:**
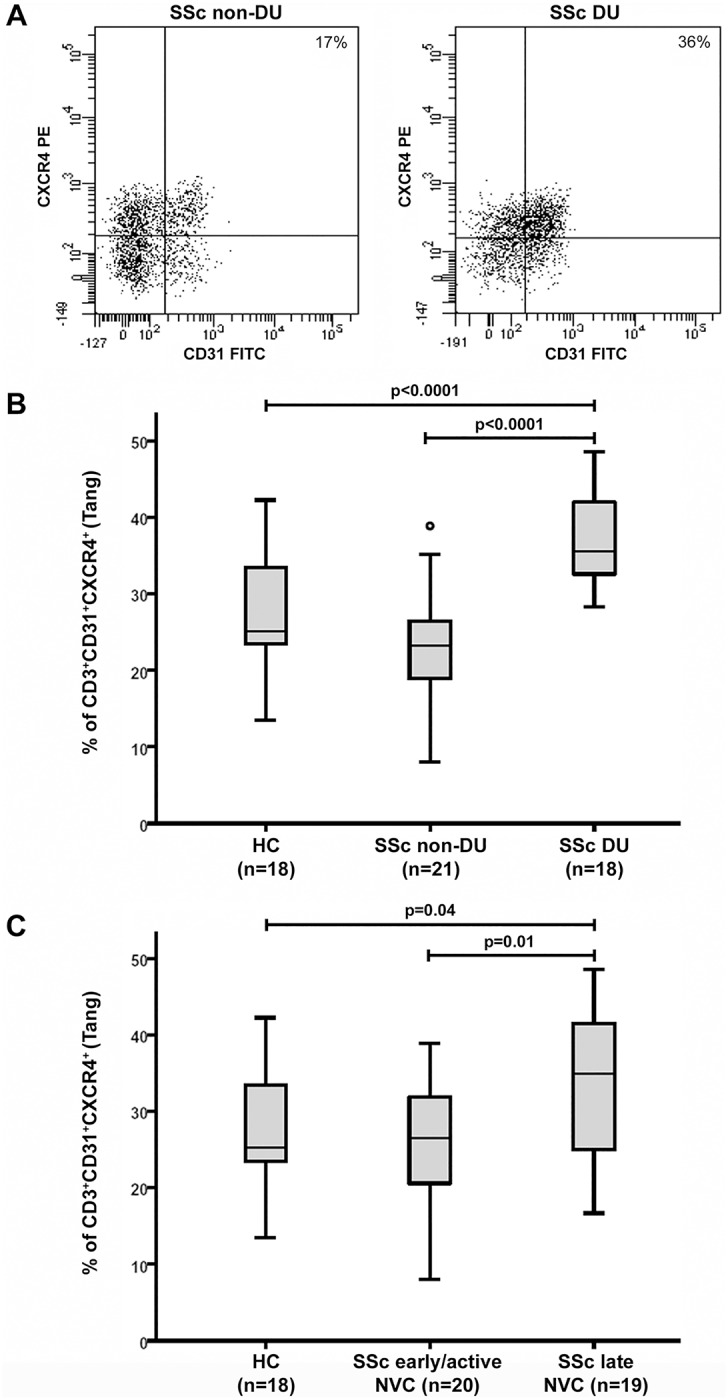
Circulating levels of angiogenic T cells (Tang) correlate with severity of peripheral vascular damage in systemic sclerosis (SSc) patients. **(A)** Representative CD31 versus CXCR4 dot plots of a SSc patient without digital ulcers (DU) and a SSc patient with DU. **(B)** Percentages of circulating CD3^+^CD31^+^CXCR4^+^ Tang cells in total CD3^+^ T cells from HC (n = 18), SSc patients without DU (n = 21) and SSc patients with DU (n = 18). **(C)** Percentages of circulating CD3^+^CD31^+^CXCR4^+^ Tang cells in total CD3^+^ T cells from HC (n = 18), SSc patients with early/active nailfold videocapillaroscopy (NVC) patterns (n = 20) and SSc patients with late NVC pattern (n = 19). Each box represents the 25th to 75th percentiles. Lines inside the boxes represent the median. Lines outside the boxes represent the 10th and the 90th percentiles. Circles indicate outliers. Differences were evaluated by Mann–Whitney U test.

However, an interesting association between Tang cell levels and the extent of peripheral microvascular damage was found. First, subgroup analysis revealed that Tang cells were significantly increased in SSc patients with DU (median 35.5, IQR 32.2−42.5) compared either with SSc patients without DU (median 23.3, IQR 18.5−26.6) or with HC (p<0.0001 for both) (Fig 2A and 2B). Furthermore, Tang cell percentage was significantly higher in SSc patients with late NVC pattern (median 34.9, IQR 25.0−42.0) than in those with early/active NVC patterns (median 26.5, IQR 20.4−32.9) and in HC (p = 0.01 and p = 0.04, respectively) (Fig 2C). On the contrary, no difference in circulating Tang cell percentages was found when comparing either SSc patients without DU or patients with early/active NVC patterns and HC (Fig 2B and 2C). No significant association was found with the other clinicodemographic and laboratory parameters of SSc patients.

As displayed in Table 2, similar results were obtained when the Tang cell population was expressed as absolute numbers of circulating CD3^+^CD31^+^CXCR4^+^ cells.

**Table 2:** Absolute numbers of circulating CD3^+^CD31^+^CXCR4^+^ angiogenic T cells (Tang) in healthy controls (HC) and systemic sclerosis (SSc) patients.

HC (n = 18)	164.5 (136.5−219.7) cells/μl
All SSc (n = 39)	187.0 (143.0−266.0) cells/μl
SSc non-DU (n = 21)	170.0 (137.5−183.0) cells/μl
SSc DU (n = 18)	263.5 (239.7−314.2) cells/μl*
SSc early/active NVC (n = 20)	174.5 (141.5−233.7) cells/μl
SSc late NVC (n = 19)	263.0 (170.0−321.0) cells/μl^§^

DU, digital ulcers; NVC, nailfold videocapillaroscopy.

Values are expressed as median (IQR).

*p = 0.006 versus HC, p = 0.002 versus SSc non-DU.

^§^p = 0.046 versus HC, p = 0.035 versus SSc early/active NVC.

Differences were evaluated by Mann–Whitney U test.

**Fig 3:**
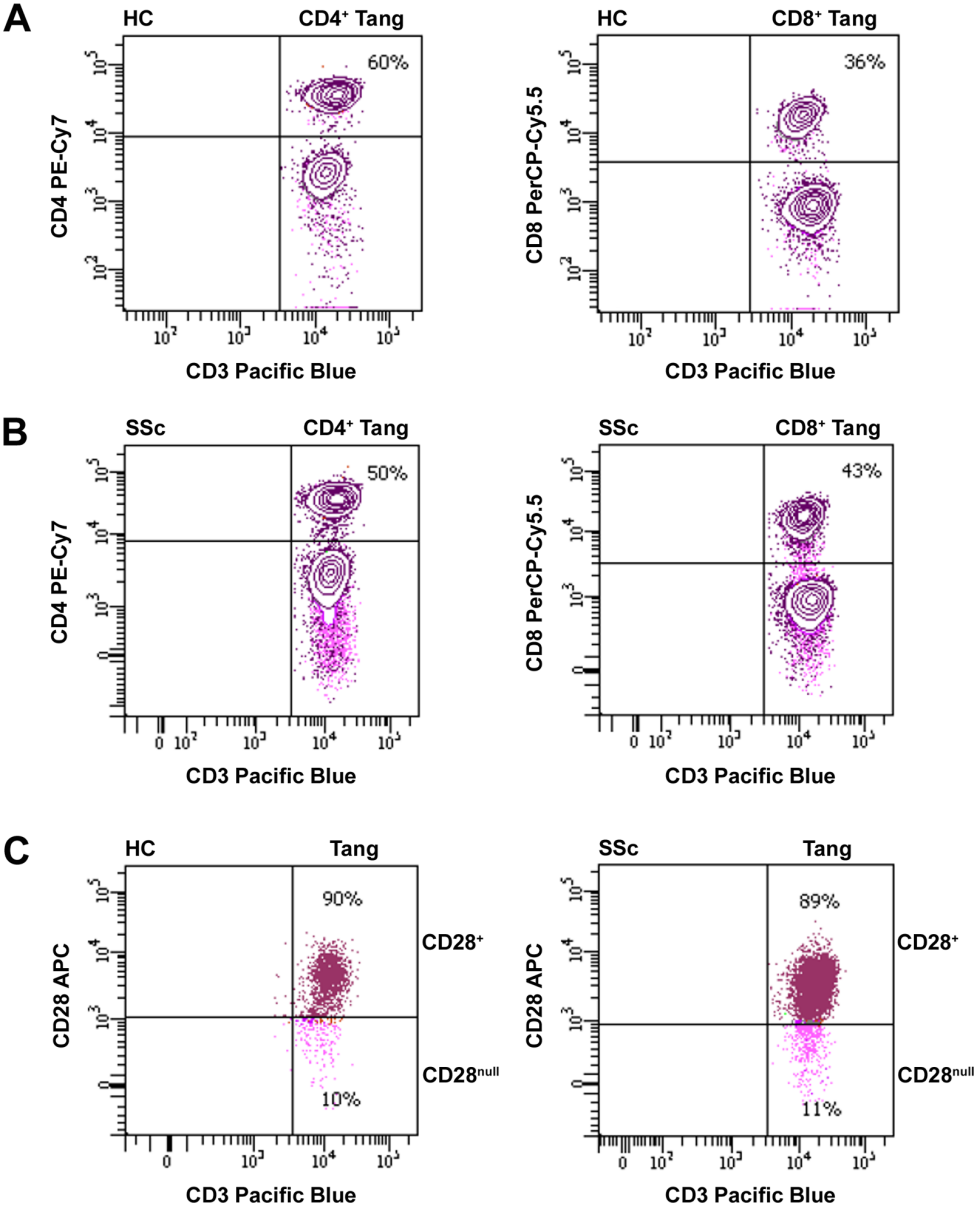
Analysis of circulating angiogenic T cell (Tang) subsets in healthy controls (HC) and systemic sclerosis (SSc) patients. **(A-C)** The proportions of CD4^+^, CD8^+^, CD28^+^ and CD28^null^ cells in total CD3^+^CD31^+^CXCR4^+^ Tang cells were assessed by flow cytometry. Representative dot plots of a HC and a SSc patient for each Tang cell subset are shown.

Moreover, we evaluated the proportions of circulating Tang cell subsets in SSc patients and HC on the basis of the expression of the CD4, CD8 and CD28 antigens (Fig 3A–3C). The percentages of CD4^+^, CD8^+^, CD28^+^ and CD28^null^ cells in total CD3^+^CD31^+^CXCR4^+^ Tang cells were similar in SSc patients and HC (Table 3). In addition, no difference in the proportions of the analyzed cell subsets in total CD3^+^CD31^+^CXCR4^+^ Tang cells was found between SSc patient subgroups according to the presence of DU and NVC patterns (Table 3).

**Table 3:** Circulating angiogenic T cell (Tang) subsets in healthy controls (HC) and systemic sclerosis (SSc) patients.

	CD4^+^	CD8^+^	CD28^+^	CD28^null^
HC (n = 18)	50.2 (43.8−59.5)	36.7 (26.1−52.8)	84.2 (77.8−89.0)	16.3 (12.2−23.4)
All SSc (n = 39)	56.0 (42.3−62.0)	36.3 (29.0−46.8)	90.4 (80.9−94.0)	10.2 (6.1−20.3)
SSc non-DU (n = 21)	55.8 (42.8−65.1)	34.2 (28.5−47.3)	90.3 (81.1−94.0)	10.1 (5.5−20.2)
SSc DU (n = 18)	57.8 (41.9−62.0)	38.7 (35.2−47.0)	87.5 (80.4−95.2)	16.4 (7.2−23.1)
SSc early/active NVC (n = 20)	54.4 (42.5−62.4)	34.9 (28.3−47.5)	89.9 (81.0−94.0)	10.2 (5.3−20.2)
SSc late NVC (n = 19)	59.6 (42.2−62.0)	37.4 (34.2−46.8)	93.0 (80.9−95.0)	14.0 (6.3−23.0)

DU, digital ulcers; NVC, nailfold videocapillaroscopy.

Values are the percentage of cells in total CD3^+^CD31^+^CXCR4^+^ Tang cells expressed as median (IQR).

**Fig 4:**
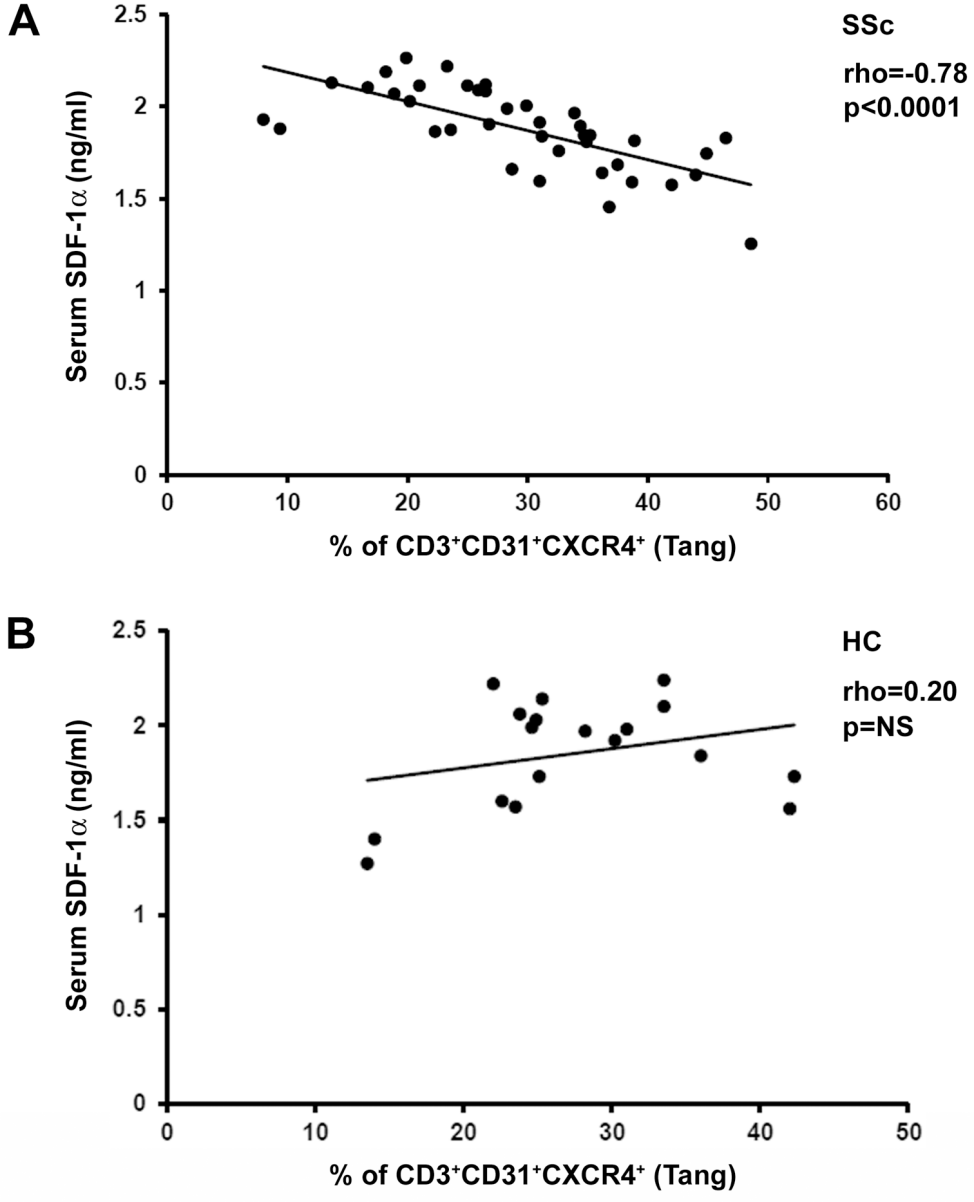
Correlation analysis of the percentage of circulating angiogenic T cells (Tang) with serum levels of stromal cell-derived factor-1α (SDF-1α) in systemic sclerosis (SSc) patients and healthy controls (HC). **(A)** In SSc patients, the percentage of CD3^+^CD31^+^CXCR4^+^ Tang cells is inversely correlated with circulating levels of SDF-1α. **(B)** No significant correlation is observed in HC. Data are shown as scatterplot, each dot representing a subject. Spearman’s rho correlation coefficient and p value are indicated. NS, not significant.

Next, we quantified circulating SDF-1α, a chemokine which may play a major role in the mobilization and trafficking of CXCR4-bearing Tang cells. In SSc peripheral blood, the percentage of Tang cells exhibited a strong inverse correlation with the levels of SDF-1α (Spearman’s rho = -0.78, p<0.0001) (Fig 4A). This result is made even more significant by the fact that such a correlation was not found in HC (Fig 4B). Interestingly, when the relationship between Tang cells and SDF-1α levels was examined in the SSc patient subgroups, a significant inverse correlation was found only in patients with DU (Spearman’s rho = -0.52, p = 0.03) and in those with late NVC pattern (Spearman’s rho = -0.74, p<0.0001).

**Fig 5:**
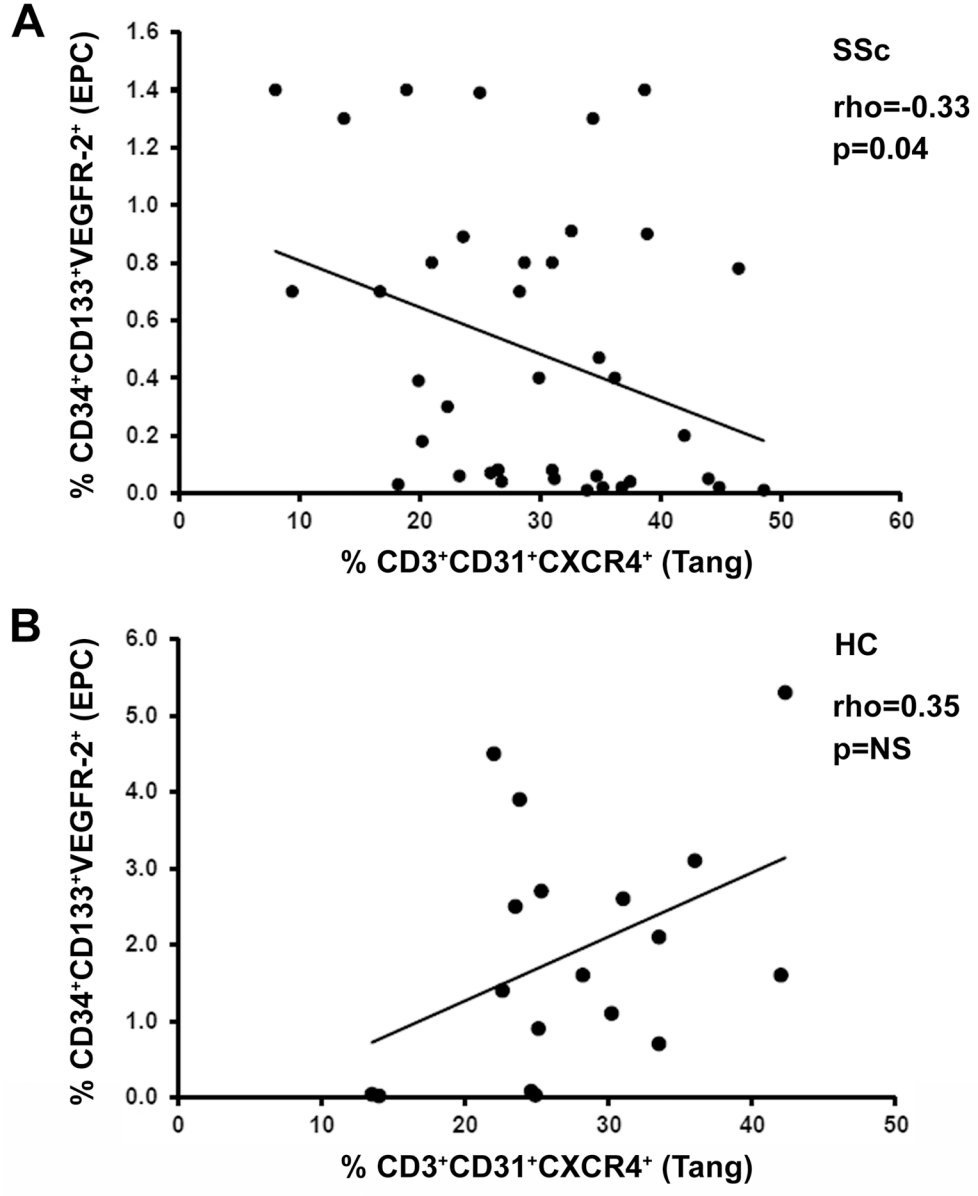
Correlation analysis of the percentages of circulating angiogenic T cells (Tang) and endothelial progenitor cells (EPC) in systemic sclerosis (SSc) patients and healthy controls (HC). **(A)** In SSc patients, the percentage of CD3^+^CD31^+^CXCR4^+^ Tang cells is inversely correlated with that of CD34^+^CD133^+^VEGFR-2^+^ EPC. **(B)** No significant correlation is observed in HC. Data are shown as scatterplot, each dot representing a subject. Spearman’s rho correlation coefficient and p value are indicated. NS, not significant.

Since previous studies have shown that Tang cells may be linked to the EPC population [17, 18], the possible relationship between circulating Tang cells and CD34^+^CD133^+^VEGFR-2^+^ EPC was explored. Consistent with previous reports [9, 10], the percentage of circulating CD34^+^CD133^+^VEGFR-2^+^ EPC in total CD34^+^ cells was significantly decreased in SSc patients (median 0.39, IQR 0.05−0.80) respect to HC (median 1.60, IQR 0.55−2.80) (p = 0.001). As displayed in Fig 5A and 5B, Tang cells exhibited a significant negative correlation with EPC levels in SSc (Spearman’s rho = -0.33, p = 0.04), but not in HC.

**Fig 6:**
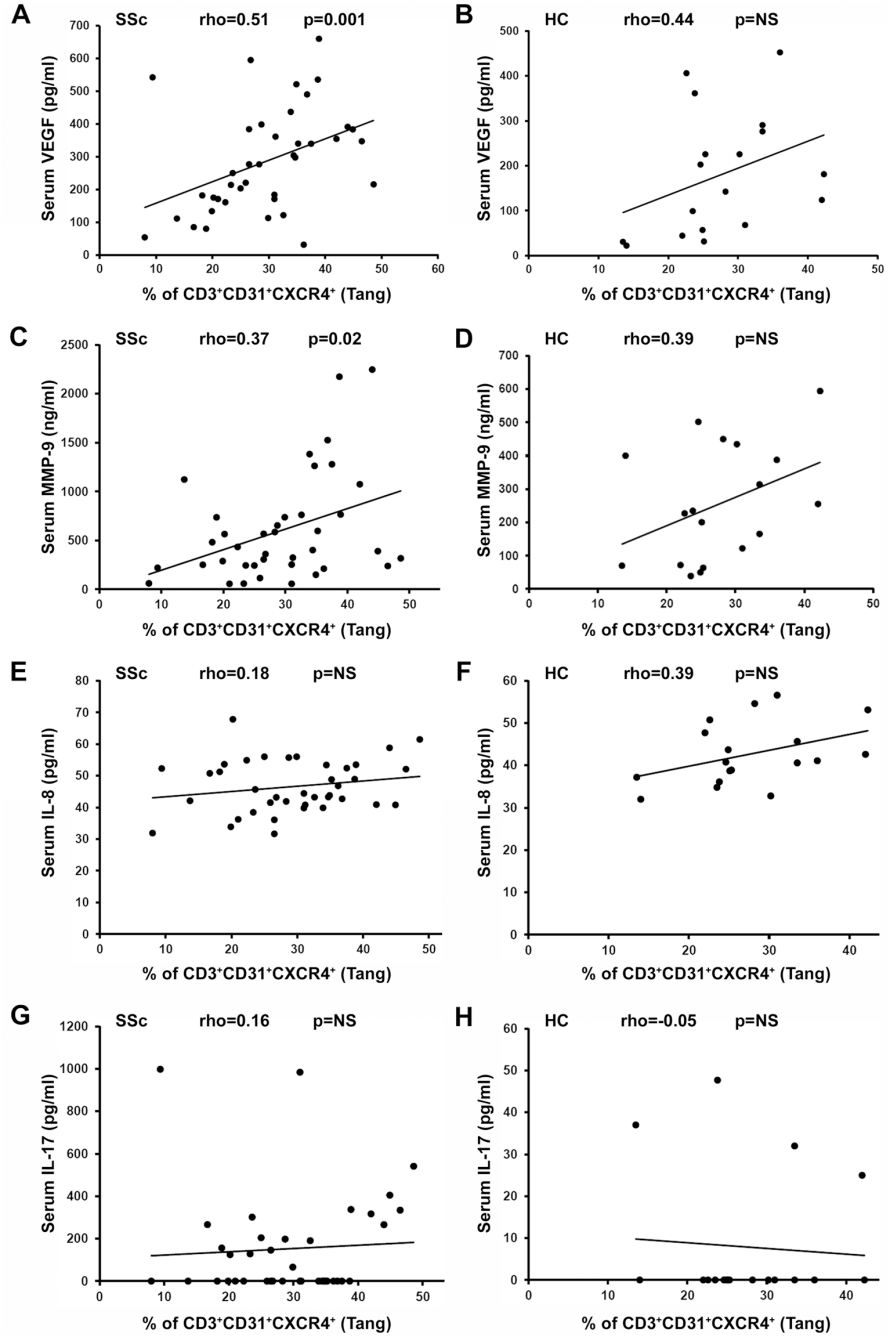
Correlation analysis of the percentage of circulating angiogenic T cells (Tang) and serum levels of proangiogenic factors in systemic sclerosis (SSc) patients and healthy controls (HC). Scatterplots of the correlation analysis between CD3^+^CD31^+^CXCR4^+^ Tang cells and serum levels of **(A, B)** vascular endothelial growth factor (VEGF), **(C, D)** matrix metalloproteinase (MMP)-9, **(E, F)** interleukin (IL)-8 and **(G, H)** IL-17 are shown. Each dot represents a subject. Spearman’s rho correlation coefficient and p value are indicated. NS, not significant.

We also investigated whether in SSc patients and HC the Tang population could correlate with circulating levels of proangiogenic factors which have been reported to be secreted by Tang cells, namely VEGF, MMP-9, IL-8 and IL-17 [17] (Fig 6A–6H). This analysis revealed that in SSc the percentage of circulating Tang cells was positively correlated with the levels of VEGF (Spearman’s rho = 0.51, p = 0.001) and MMP-9 (Spearman’s rho = 0.37, p = 0.02) (Fig 6A and 6C), but not with the levels of IL-8 and IL-17 (Fig 6E and 6G). None of the analyzed factors was found to be significantly correlated with Tang cells in HC (Fig 6B, 6D, 6F and 6H).

**Fig 7:**
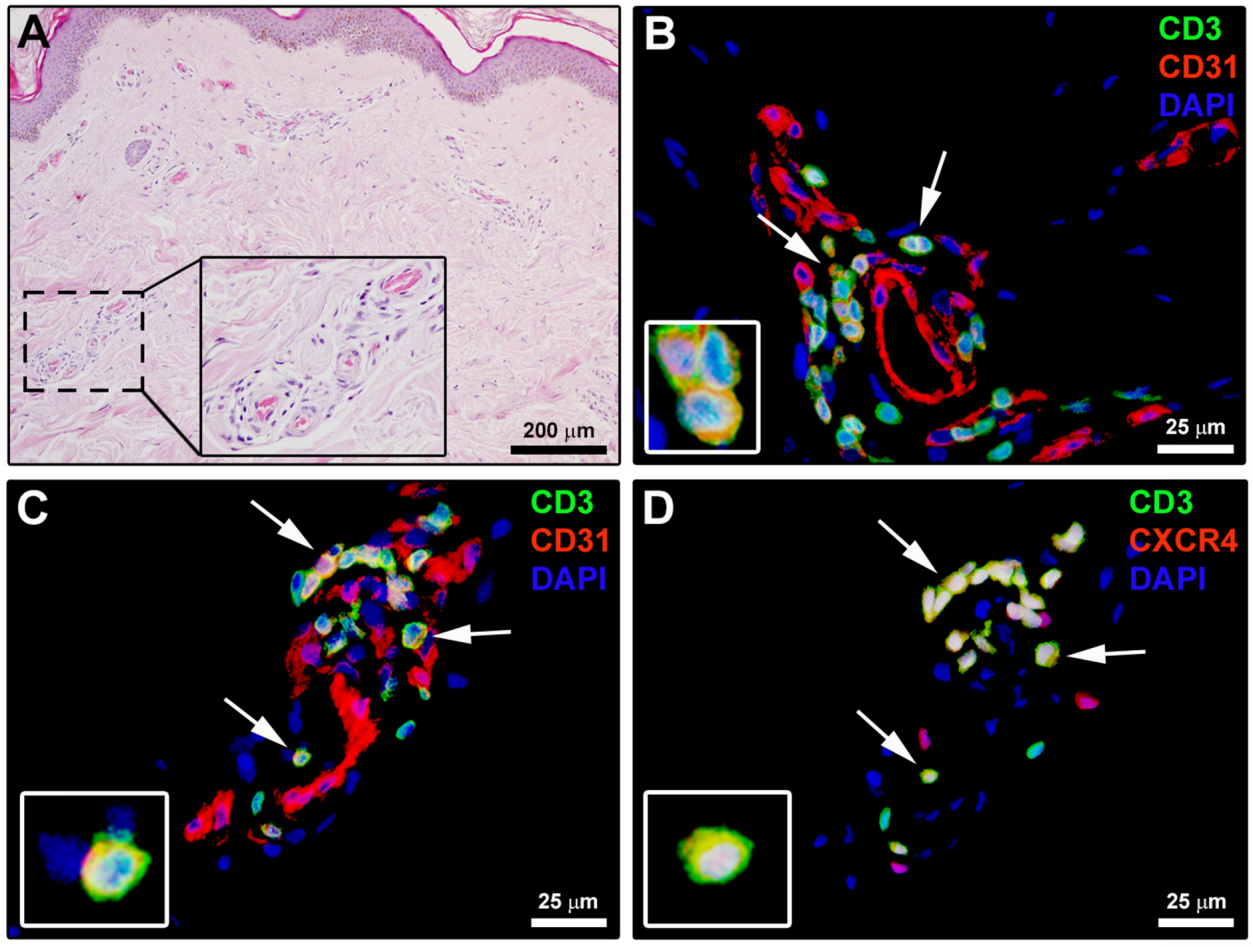
Angiogenic T cells (Tang) are present in systemic sclerosis (SSc) dermal perivascular inflammatory infiltrates. **(A)** Representative microphotograph of skin sections from SSc patients stained with hematoxylin and eosin. Perivascular inflammatory cells from the boxed area are shown at higher magnification in the inset. **(B)** Representative fluorescence microphotograph of skin sections from SSc patients double immunostained for the pan-T lymphocyte marker CD3 (green) and CD31 (red), and counterstained with 4′,6-diamidino-2-phenylindole (DAPI; blue) for nuclei. Arrows indicate CD3^+^CD31^+^ T lymphocytes. **(C, D)** Representative fluorescence microphotographs of serial skin sections from SSc patients double immunostained for CD3 (green) and CD31 (red; **C**) or CXCR4 (red; **D**). Arrows indicate CD3^+^CD31^+^CXCR4^+^ T lymphocytes (Tang). Insets: higher magnification views of immunopositive lymphocytes from the respective panels. Scale bars are indicated in each panel.

Finally, the possible presence of Tang cells in the biopsies of the forearm skin affected by the disease was investigated by immunofluorescence (Fig 7A–7D). As displayed in Fig 7B, CD3^+^CD31^+^ T lymphocytes were frequently observed in SSc dermal perivascular inflammatory infiltrates, while they could not be detected in HC skin. Moreover, CD3/CD31 and CD3/CXCR4 double immunofluorescence performed on serial skin sections clearly demonstrated the presence of perivascular CD3^+^CD31^+^CXCR4^+^ Tang cells in SSc dermis (Fig 7C and 7D).

## Discussion

This is the first study investigating the possible involvement of the recently identified Tang cell population in SSc. Our findings demonstrate that Tang cells are selectively expanded in the circulation of SSc patients displaying severe peripheral vascular complications like DU. In fact, patients with advanced derangement of the dermal capillary network and DU had circulating levels of Tang cells significantly higher than HC, both in absolute numbers and as a percentage of T cells. On the contrary, Tang levels did not differ between HC and SSc patients with moderate capillary damage and lack of DU. Thus, our data suggest that circulating Tang cells might represent a novel biomarker closely reflecting the severity of SSc-related peripheral vasculopathy.

The maintenance of endothelial homeostasis and the formation of new blood vessels are of paramount importance in the control of human health and disease. They are finely tuned and controlled by a complexity of soluble and cellular components. In this context, Tang are a unique T cell subset endowed with proangiogenic functions capable of sustaining endothelial repair by interacting with EPC and endothelial cells [17–20]. Tang, first described by Hur *et al*. in 2007 [17], are CD3^+^CD31^+^CXCR4^+^ cells required for *in vitro* colony formation and differentiation of early hematopoietic EPC. Furthermore, it appears that Tang cells may enhance endothelial proliferation and functions by either cell contact-dependent or paracrine mechanisms [17]. In particular, Tang secrete a wide array of proangiogenic factors, including VEGF, IL-8, IL-17, granulocyte-colony stimulating factor, and MMP-9 [17]. Altogether, these properties give Tang the ability to stimulate angiogenesis *in vitro* and promote neovascularization of ischemic tissues *in vivo*, as demonstrated in a hind limb ischemia experimental model [17].

Growing evidence indicates that impaired angiogenesis and vasculogenesis critically contribute to the pathogenesis and clinical manifestations of SSc [2, 9, 11, 12]. An abnormal expression of several proangiogenic and angiostatic factors has been reported in SSc tissues and peripheral blood [7, 12, 25, 26]. Moreover, a number of *in vitro* and *in vivo* studies showed that either dermal microvascular endothelial cells or bone marrow-derived circulating EPC are defective in SSc [8–16]. The present findings provide the first evidence that Tang cells may be part of this complex scenario. Indeed, differential percentages and absolute numbers of circulating Tang cells were detected in SSc patients according to the severity of peripheral vasculopathy. Furthermore, our immunohistologic analyses revealed that Tang cells are present in perivascular inflammatory infiltrates of early SSc skin lesions. It appears that Tang are endowed with a high capacity of adhesion to endothelial cells and transendothelial migration through the endothelial junctions using CD31, as well as a great capacity to invade the ischemic tissue using MMP-9 [17]. It has also been postulated that Tang cells expressing high levels of CXCR4 home to areas of ischemia where SDF-1 level is high [17]. Of note, previous studies from our group have shown that dermal expression of SDF-1 is strongly increased in the early phase of SSc [27]. In addition, in our patients the percentage of Tang cells was inversely correlated to the circulating levels of SDF-1α. Thus, in SSc the SDF-1/CXCR4 axis might play a major role in the homing of Tang cells to the affected skin.

The expansion of Tang cells observed in SSc patients with significant microvascular involvement and reduction of the blood flow might be a reaction to an inefficient angiogenesis and EPC function. This hypothesis is supported by the evidence that the levels of Tang cells were inversely correlated with those of EPC in the circulation of SSc patients. Furthermore, in SSc patients circulating Tang levels exhibited a positive correlation with the levels of VEGF and MMP-9, two soluble mediators that have been implicated in SSc-related angiogenic disturbances and that can be secreted even by Tang themselves [11, 12, 17, 28].

In line with our findings, ANCA-associated vasculitis patients with relapsing disease course showed an expansion of circulating Tang [20]. Moreover, another study reported that the percentage of circulating CD8^+^ Tang cells was significantly increased in SLE patients when compared to HC [19]. Conversely, lower Tang cell numbers have been associated with vascular disease in RA and hypertensive patients [18, 29]. Of note, a very recent study revealed the existence of two different subsets of Tang cells based on CD28 expression, which may be endowed with opposite functions [30]. In particular, it appears that the CD28^null^ Tang subset displays a senescent phenotype and might exert cytotoxic and inflammatory rather than protective effects on endothelial cells [30, 31]. Interestingly, the percentage of CD28^null^ Tang cells was found to be especially increased in patients with traditional cardiovascular risk factors and patients with SLE, but not in RA patients [30]. However, when we investigated Tang cell subsets on the basis of the expression of CD4, CD8 and CD28 antigens, we could not find any significant difference in the proportions of CD4^+^, CD8^+^, CD28^+^ and CD28^null^ Tang cells either between SSc patients and HC or between SSc patient subgroups stratified according to the severity of peripheral vasculopathy. Thus, the expansion of Tang cells observed in SSc patients with more severe peripheral vascular complications seems not attributable to a specific Tang subpopulation.

Our study has some limitations. First, the study design is cross-sectional and therefore prospective analyses will be required to ascertain whether the proportion of circulating Tang cells may vary in SSc patients with progression of peripheral vascular damage. Furthermore, our findings need to be replicated in larger and independent cohorts of SSc patients. Notwithstanding these limitations, the strength of our study remains the novelty of our findings in a well-characterized and homogeneous group of SSc patients.

## Conclusions

In summary, we found that Tang cells relate to the severity of peripheral vascular disease in SSc patients. Considering the relevance of Tang cells in the control of angiogenesis and endothelial and EPC homeostasis, it may be of interest to further investigate the feasibility of these cells as a potential surrogate biomarker of cardiovascular risk factors and therapeutic target for SSc patients. Finally, further studies are required to clarify the function of Tang cells and investigate the mechanisms responsible for their change in SSc.
